# Real-Time Detection of Cook Assistant Overalls Based on Embedded Reasoning

**DOI:** 10.3390/s21238069

**Published:** 2021-12-02

**Authors:** Qinghua Sheng, Haixiang Sheng, Peng Gao, Zhu Li, Haibing Yin

**Affiliations:** School of Electronics and Information, Hangzhou Dianzi University, Hangzhou 310018, China; sheng7@hdu.edu.cn (Q.S.); haixiangsheng@hdu.edu.cn (H.S.); gaopeng@hdu.edu.cn (P.G.); lz1126@hdu.edu.cn (Z.L.)

**Keywords:** edge computing, edge intelligence, Hi3559, overall recognition, embedded

## Abstract

Currently, the target detection based on convolutional neural network plays an important role in image recognition, speech recognition and other fields. However, the current network model features a complex structure, a huge number of parameters and resources. These conditions make it difficult to apply in embedded devices with limited computational capabilities and extreme sensitivity to power consumption. In this regard, the application scenarios of deep learning are limited. This paper proposes a real-time detection scheme for cook assistant overalls based on the Hi3559A embedded processor. With YOLOv3 as the benchmark network, this scheme fully mobilizes the hardware acceleration resources through the network model optimization and the parallel processing technology of the processor, and improves the network reasoning speed, so that the embedded device can complete the task of real-time detection on the local device. The experimental results show that through the purposeful cropping, segmentation and in-depth optimization of the neural network according to the specific processor, the neural network can recognize the image accurately. In an application environment where the power consumption is only 5.5 W, the recognition speed of the neural network on the embedded end is increased to about 28 frames (the design requirement was to achieve a recognition speed of 25 frames or more), so that the optimized network can be effectively applied in the back kitchen overalls identification scene.

## 1. Introduction

Food safety problems are prevalent around us. According to the World Health Organization, about 600 million people worldwide get sick and 420,000 people die of eating contaminated food every year, causing a loss of 33 million healthy life years [1]. Since 2014, CFDA has carried out the movement of “transparent kitchen and stoves”, with an aim to make public the food processing process through video display, partition of short wall, open kitchen and other forms, and place the key parts and links of catering service under the social supervision. We can collect kitchen data via cameras, but how to correctly recognize and supervise these data has become a problem. The traditional way of labor supervision requires a lot of time and labor costs, resulting in unsatisfactory effects. Therefore, this paper hopes to use deep learning to automatically complete the detection process of whether the staff entering the kitchen are wearing kitchen clothes, by computer equipment.

Video processing technology with deep learning as the core has been fully applied in face recognition and vehicle recognition [2,3,4]. The current mainstream deep learning solutions are deep learning reasoning through high-performance GPU and then deployment to the local end users using cloud servers. However, in the actual use case scenarios, this scheme is faced with high costs, a high power consumption and a limited bandwidth. We therefore try to perform reasoning calculations locally using embedded devices. This scheme can effectively reduce the device cost and bandwidth requirements while controlling the power consumption of the device. At present, embedded devices cannot directly meet the requirements for deep learning due to their weak computational capabilities. This paper aims to design a solution for cook assistant overalls recognition on an embedded device based on YOLO V3 and provide a feasible technical scheme for the embedded application of deep learning [5]. The current common acceleration schemes are roughly divided into three following categories:

(1) A more lightweight network model is designed. The model can find a better balance between precision and memory consumption. For example, the MobileNet model is in a new lightweight model structure [6]. VGG-16 network performance can be implemented with a 10-fold decrease in the calculated quantity.
(2) Software optimization techniques and mathematical methods are used to trim the network, so as to reduce the network calculation and memory requirements. For example, He Yang’s team reduced the calculation amount of ILSVRC-2012 by over 40% on ResNet-50 through progressive soft-filtering pruning, with 0.14% reduced accuracy [7].
(3) An hardware accelerator customized for deep learning algorithm or FPGA is used to perform the high-concurrent operations and complete the acceleration process. For example, Kang’s team won the first prize of the LPIRC competition through optimization algorithm using NVidia TX2 [8]; Ma’s team increased the reasoning speed of tiny-yolo to 20 frames using FPGA [9,10].

This paper proposes the scheme of pipelining [11,12] and parallel processing on the basis of integrating the above schemes, with an aim to improve the network reasoning speed from the perspective of improving the system hardware resources utilization.

The main contribution and work contents proposed in this paper are:

(1) Deep learning is applied to the kitchen target detection to realize the detection of whether the workers entering the kitchen are wearing kitchen overalls. In addition, the whole system is finally deployed on low-power embedded products, which greatly reduces the cost of equipment and power consumption required for traditional deep learning target detection, so that deep learning can be applied to more use case scenarios.
(2) This paper introduces the idea of software pipelining in deep learning to transform the network reasoning process from single tasks implemented step by step to different stages of multiple tasks, and thus greatly improve the utilization rate of hardware resources and the recognition speed of the system.
(3) Speed matching between the anterior and posterior networks is achieved through unbalanced segmentation of the model and multithreaded optimization [13]. After the introduction of the circle buffer, it can ensure the error of a single task does not affect other tasks.
(4) The goal of this scheme is to complete the intelligent processing of video data on the “edge side”. The videos of multiple cameras on the local switch can be detected through the embedded reasoning card to recognize whether the chefs correctly wear the overalls. The upper computer can display the recognition results, and set the recognition rate of the card, the threshold and the number of cameras.

The rest of this paper is arranged as follows: Section 2 introduces the implementation process of the system and each part; Section 3 introduces the deep learning acceleration methods based on the Hi3559A embedded processor; Section 4 displays the experimental processes and results; Section 5 gives the conclusions. 

## 2. System Scheme

### 2.1. Scheme Introduction

**Figure 1:**
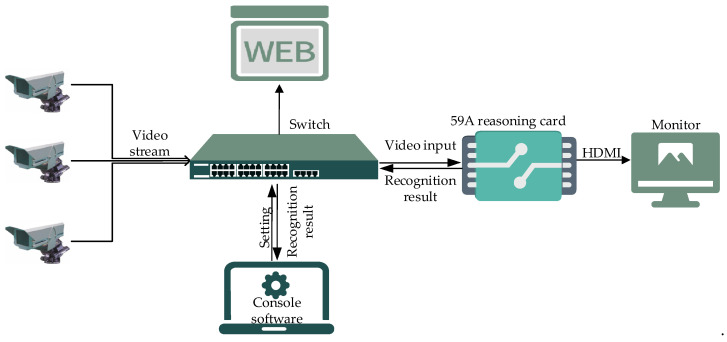
System scheme.

The system scheme is shown in Figure 1, in which the reasoning card is connected to the local switch and the console software controls the reasoning card. The reasoning card may decode/recognize/code the video streams and display the coded detection results on the monitor. The results are uploaded to the front-end WEB system via JSON coding.

### 2.2. Embedded Deployment Scheme

**Figure 2:**
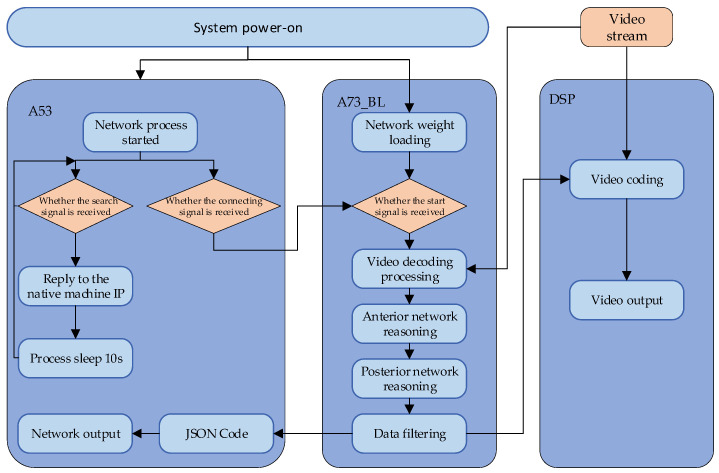
Block diagram for embedded software.

The block diagram for embedded software is shown in Figure 2. Through multi-core deployment and collaboration, the overall computing power and resource throughput rate are significantly improved. In Figure 2, an A53 kernel is responsible for the network process, which can complete the data interaction and communication with the console; an A73 kernel is responsible for the core scheduling and controlling the coordination between image coding/decoding and pipelining; a DSP kernel is responsible for the video coding/output.

### 2.3. Interface of Console Software

**Figure 3:**
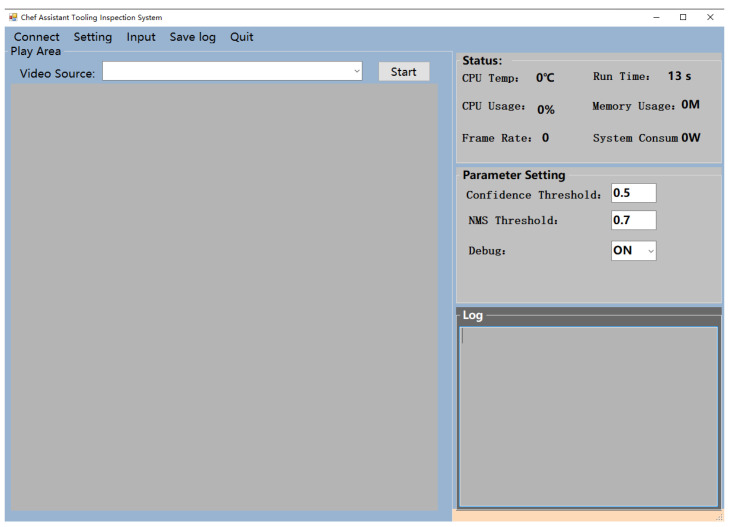
Interface of console software.

The interface of console software is shown in Figure 3: after the system start, the console will search for the 59A reasoning card device in the local switch by means of multicast and add the searched devices to the menu bar. Then, one can search for the input camera and video sources, and finally set the parameters to start the device.

## 3. Core Acceleration Method

### 3.1. Introduction of Pipelining Technology

**Figure 4:**
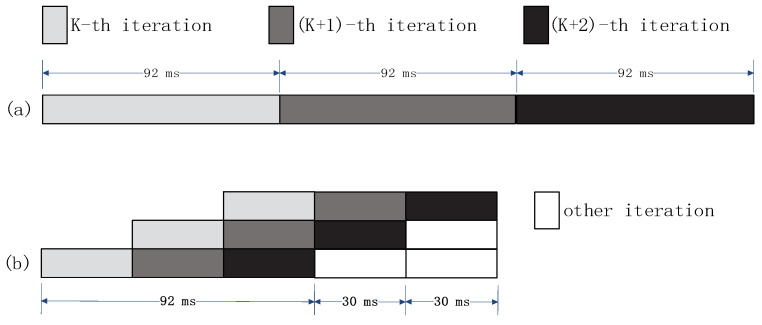
Schematic diagram of pipelining.

Pipelining refers to a parallelization means of superposing multiple instructions in an instruction cycle. The technology is generally applied in general computing processors. Pipelining can reduce the waiting time of the CPU and achieve the continuous use of the CPU resources, thus improving the overall utilization of the CPU. See Figure 4 for a diagram of pipelining.

The scheme of this paper draws lessons from the idea of pipeline technology in CPU, By dividing the network and task, the whole process is divided into graph acquisition, decoding, reasoning, post-processing, video coding output, etc., which greatly improves the utilization rate of hardware resources and finally recognizes an image frame in a reasoning cycle. 

Figure 4a shows that the cycle time of a single cycle of processing is 92 ms. After the idea of pipeline is introduced in Figure 4b, the processing cycle is compressed to about 30 ms due to the reduction of the waiting time between the hardware.

The advantages of introducing pipelining are:

(1) It greatly improves the utilization rate of hardware resources, and realizes the parallel execution of multiple tasks of different stages at the same time;
(2) Although pipelining operates multiple network data at the same time, the network only needs to load once, which greatly reduces the memory occupation compared with traditional multi-operators. This is very important for embedded devices;
(3) Pipelining with a buffer zone can effectively reduce the processor’s idle time through reasonable task distribution, without additional multi-process time consumption;
(4) Pipelining is more conducive to protecting the relationship between frames before and after pictures. The order of data processed is synchronized, without additional thread synchronization tools.

### 3.2. Network Cutting

**Figure 5:**
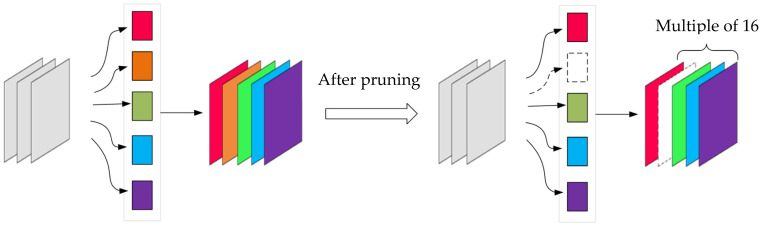
Schematic diagram of network pruning.

Complex models generally have redundant parameters to ensure a balance between performance and robustness. These redundant parameters can be cropped by tools. The model parameters are reduced by cutting out less weighted link layers to achieve model compression [14,15]. In this cutting scheme, the number of network dimensions is a multiple of 16 through additional inducing factors, which can effectively read the efficiency and computing performance using HiSilicon memory. Figure 5 shows the dimension changes before and after pruning.

L1 regularization is used for thinning training in the training process. Neural networks have many parameters approaching 0 after L1 regularization training. These parameters can make small contributions to the final result. The spinning training and pruning are combined in this paper. On the one hand, pruning is used to reduce the unimportant link layer, and on the other hand, the weights are induced in training to make the network sparser.

After testing, the accuracy of the network recognition was significantly reduced when the pruning ratio is greater than 50%. Through multiple experimental comparisons, the pruning ratio of 40% was selected, reducing the calculation significantly to 58.59% of the original and the accuracy by only 0.19%. It can improve the reasoning speed of the network effectively while ensuring accuracy.

### 3.3. Model Segmentation and Quantization

A Hi3559Av100 with two neural network inference engines (NNIEs) is used as the main control chip. This scheme tries two deployment modes, parallel deployment and serial deployment. Parallel deployment means that a complete network is run separately in each NNIE and the input images are assigned to the two networks alternately. Serial deployment is the splitting of a network into two halves, with each NNIE running only a portion of the entire network. In theory, both schemes can achieve twice the speed of a single NNIE. However, through comparative experiments, we proved that the parallel deployment scheme was limited by the memory bandwidth and could not unlock the full computing power. So we adopted a serial deployment scheme as the preliminary deployment scheme.

The model quantification refers to the technical coding means that are used to reduce the memory space occupied by the parameters in the model, thus achieving model compression. Because HiSilicon needs to quantize the network structure (8 bits) in the process of network model transformation, it will consume a large number of computations in the process of quantification. Therefore, to balance the anterior network and posterior network, the reasoning time of the anterior network needs to add the calculation time of the quantification process. After comparing various segmentation schemes, we finally divided the whole network model into two unbalanced sub-networks, which ran independently in different network acceleration engines. The quantification Formula (1) can be obtained from the data fitting, as shown below:

(1) $$\left\{\begin{array}{l} i=round(\ln(256*\frac{x}{|data{|}_{max}})*\frac{128}{\ln(256)}),x\geq 0\\ i=-round(\ln(-256*\frac{x}{|data{|}_{max}})*\frac{128}{\ln(256)}),x<0 \end{array}\right\}$$

The *x* in the formula represents the input floating-point number, i represents the quantized value after conversion, and the data represent the set of input data. In order to ensure that the input of the ln function is a positive value, we consider whether *x* is greater than 0 or not.

**Figure 6:**
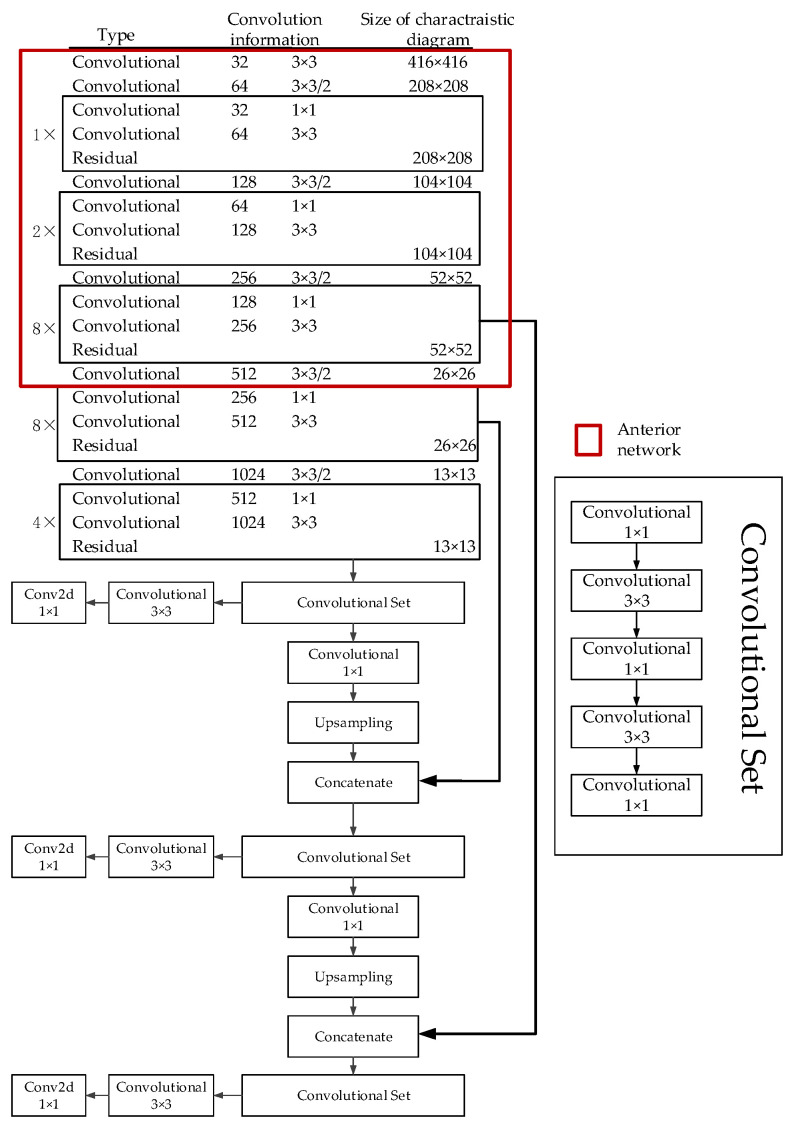
Network structure and network split results.

According to the dimension of the input image, the calculation amount of the quantification process is about 7 GFLOPS. Therefore, an unbalanced segmentation is required to balance the anterior network and posterior network. The segmentation results are shown in Figure 6 (the anterior network shown in red circle).

Following the segmentation in Figure 6, the network calculation and data size are shown in Table 1.

### 3.4. Fixed-Point Data Flow

In most CPUs, calculating with floating-point numbers is much slower than with integers. Taking the Hi3559 series of chips as an example, the inner NNIE core calculates the fixed-point data much faster than the floating-point numbers. Therefore, the data need to complete the transformation from floating-point number to fixed-point number before entering the computer core [16].

In the forward reasoning process of the network, only the forward multiplication and addition calculation is required because the weights are fixed, so the error after the fixed-point conversion does not increase exponentially. The results of statistics and testing on the server show that the network weights and activation values of YOLOv3 are generally distributed between ±20. There is no significant accuracy difference on the COCO dataset after 12-bit fixed data, except the confidence is a few percentage points lower relative to the original data [17]. Therefore, it is somewhat feasible to use fixed-point data for network reasoning.

**Figure 7:**
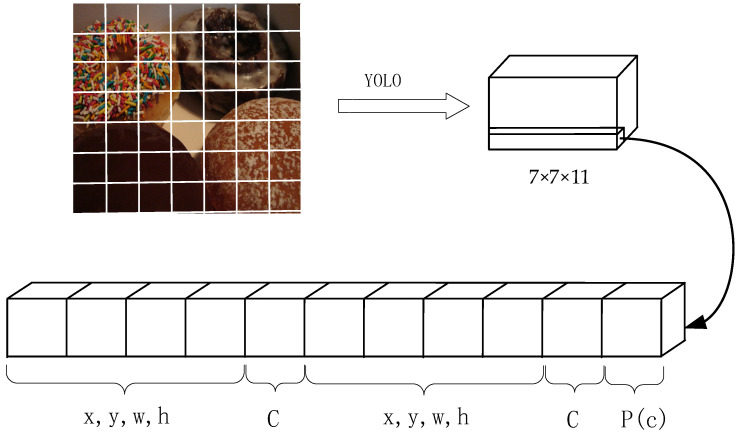
Schematic diagram of YOLO network output structure.

The coding information of the network output results at certain dimensions is shown in Figure 7. Supposing two tensors are adopted in the network, *x* and *y* are offsets of the boundary center box relative to the upper left corner of the grid; *w* and *h* are the width and height of the bounding box, respectively. C is used to determine the credibility of the target, and P(C) to determine the probabilities that the target belongs to a certain class. Formulas (2)–(5) represent the transformation between the relative and the absolute coordinates.

(2) $$b_{x}=sigmoid(t_{x})+c_{x}$$

(3) $$b_{y}=sigmoid(t_{y})+c_{y}$$

(4) $$b_{w}=p_{w}e^{t_{w}}$$

(5) $$b_{h}=p_{h}e^{t_{h}}$$

where $c_{x}$ and $c_{y}$ represent the position of the upper left corner of the grid cell relative to the entire picture, $t_{x}$ and $t_{y}$ represent the offsets of the center position of the recognition target relative to the upper left corner of the current grid cell. In order to prevent the calculated center coordinates from exceeding the range of the current grid cell, we need to normalize $t_{x}$ and $t_{y}$ using the sigmoid function; $b_{x}$ and $b_{y}$ are the absolute coordinates of the target center position. $p_{w}$ and $p_{h}$ represent the height and width of the anchor box, respectively; $t_{x}$ and $t_{y}$ represent the width and height, respectively, directly predicted by the bounding box; $b_{w}$ and $b_{h}$ indicate the actual width and height of the forecast, respectively.

The network output result is a fixed-point number, and the process of network screening and NMS in the traditional post-processing process requires a floating-point number result. In this design, the process of converting the network output result into a floating-point number is cancelled. By reconstructing the filtering algorithm, fixed-point numbers can be used to complete data filtering and accuracy calculations, which greatly reduces the complexity of the calculations.

The pseudo-code of Algorithm 1 shows the process of filtering network results after reloading.
**Algorithm 1** Data filtering and calculation1: tensor parseYolov3Feature(Tensor features, conf_threshold) 2:    conf_threshold = anti_sigmoid(conf_threshold) << 12 3:    for feature in features do 4:        confidence = feature.data[c] 5: if (feature.confidence >= conf_threshold) then 6:          (tx, ty, tw, th, tc) = feature.data[(x, y, w, h, c)] * 1.0f/4096 7:           (x, y, w, h) = computers_box(tx, ty, tw, th) 8:          Class_confidences[i] = feature.data[conf] * 1.0/4096 9:          Softmax(class_confidences) 10:          box = (class, confindences, x, y, w, h) 11:          Boxes.push_back(box) 12:    return Boxes 

### 3.5. Multithreaded Optimization

**Figure 8:**
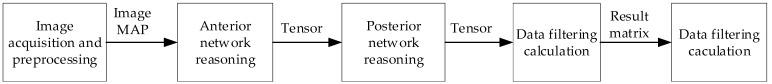
Schematic diagram of the task data flow.

After the overall task is segmented into tasks independent of each other, the above combined tasks are deployed multithreaded on the embedded master control. The thread access safety is guaranteed through the mutual exclusion and synchronization mechanism of the threads. The data flow diagram for the tasks in this design is as shown in Figure 8.

#### 3.5.1. Shared Memory in the Critical Zone

There is a single-directional data interaction between the individual tasks. Currently, the multithreaded synchronization and mutual exclusion mechanism includes signal volume, read/write lock, conditional variable, mutual exclusion lock, spin lock, etc. As shown in Figure 8, the data volume of the system is huge and the overall instantaneity of the system should be guaranteed by the stable data processing time. So we chose a shared memory method for data exchange.

**Figure 9:**
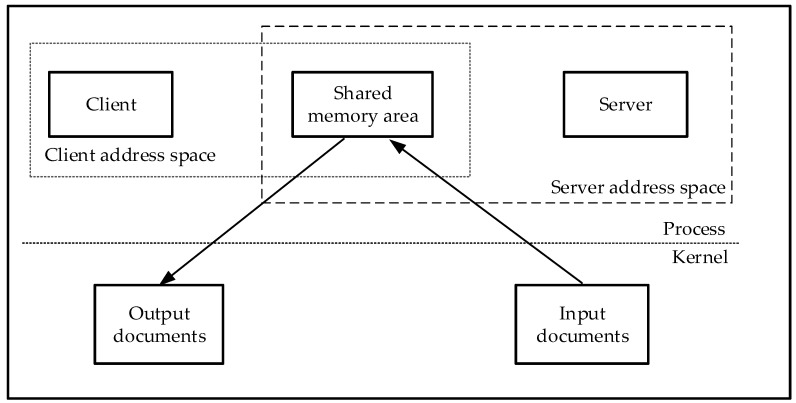
Shared memory between the processes.

Shared memory refers to common access to multiple threads by mapping a piece of memory that can be accessed jointly by multiple processes, which is a resource in the critical region [18,19]. As shown in Figure 9, the shared memory may communicate a large amount of data without the additional replication of data. Shared memory is done directly in the memory space, atomicity between threads cannot be guaranteed. Therefore, the shared memory itself does not provide a solution for process synchronization, and needs to solve the problem of inter-thread synchronization with other synchronization tools.

#### 3.5.2. Introduction of a Circle Buffer

The network processing time of each pipelining task is affected by the working environment, the number of targets in the picture, and the quality of the input image. Some unexpected factors will lead to a slow execution speed and block the pipelining, thus affecting the overall speed of the system, and even leading to the reading of dirty data or access to invalid memory. In all the links, the image acquisition is the most vulnerable to the network environment, the reading speed of the storage media and other factors. Therefore, it is necessary to add an appropriate buffer zone to the original scheme to ensure the overall stability of the system.

**Figure 10:**
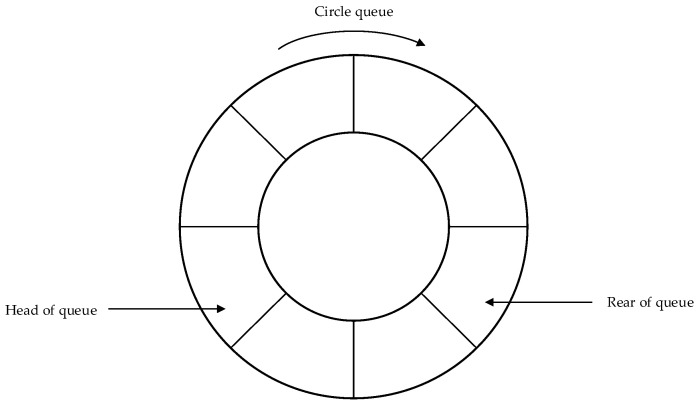
Schematic diagram of the circle queue.

The entire reasoning process requires a large amount of data and frequent requests to release memory. The linear buffer may produce an amount of memory fragmentation. In order to avoid system collapse due to excessive memory fragmentation, this scheme establishes a circular buffer pool mechanism that can effectively avoid the frequent creation, allocation and release of memory in the linear buffer. As shown in Figure 10, the circle buffer usually sets read/write indicators after the application for memory. The write pointer points to the next available location in the current memory segment. When the current location address exceeds the requested memory area, the write pointer will return to the start position of the application area. The read pointer reads the valid data in memory along the route of the write pointer. This design is a one-way communication without the introduction of multiple pointers, so mutual exclusion lock is not required to ensure the memory safety.

Using the circle queue buffer, the system’s memory increased by 12% and the speed increased to about 28 frames. Moreover, it was more stable during a long period of work. After six hours of testing, no access to invalid memory occurred.

## 4. Experiments

Hi3559Av100 is selected as the embedded main control chip. The data set was a custom dataset for cook assistant overalls. An Intel i7-6850K CPU @ 3.60GHz experimental platform was used in the training process with an NVIDIA GTX1080ti*3; the operating system was Ubuntu 18.04.

### 4.1. Experimental Environment Preparation

Numerous parameter computations are required for deep learning network training. This part needs to be placed on a server with certain operational capabilities. The computer configuration used for this training is shown in Table 2.

The network training process relied on the darknet framework [20]. The cafe framework [21] was required for the model transformation. After installing the above environment correctly, the upper sampling layer needed to be added manually in cafe to ensure the correct model conversion.

### 4.2. Dataset Used for the Experiment

**Figure 11:**
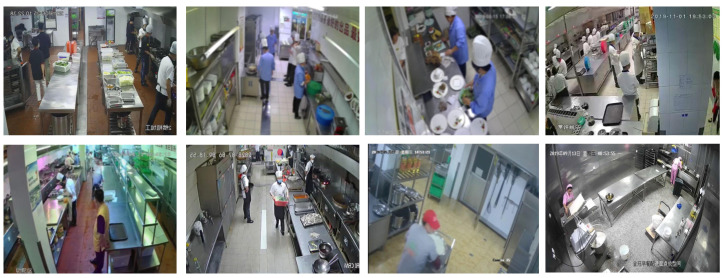
Display of some images from the dataset.

The dataset used herein is a self-built kitchen overall dataset. A total of 33,254 monitoring pictures were collected in 37 different scenes, which were called the cook dataset. There were eight categories in the dataset, i.e., 0—gray, 1—black, 2—white, 3—blue, 4—red, 5—yellow, 6—pink and 7—others. Subsequently, the dataset was expanded using tilt, mirroring, Gaussian blur and histogram equalization. As a result, there were 43,255 pictures in the final dataset, as shown in Figure 11.

### 4.3. Network Result Validation

To verify the overall identification algorithm, we first needed to train the weights of the YOLO network. In this paper, the ratio of the training, validation and test sets was set to 8:1.5:0.5 and the number of images were 34,604, 6488 and 2163, respectively.

The hyper-parameter settings for the YOLO network are shown in Table 3.

**Figure 12:**
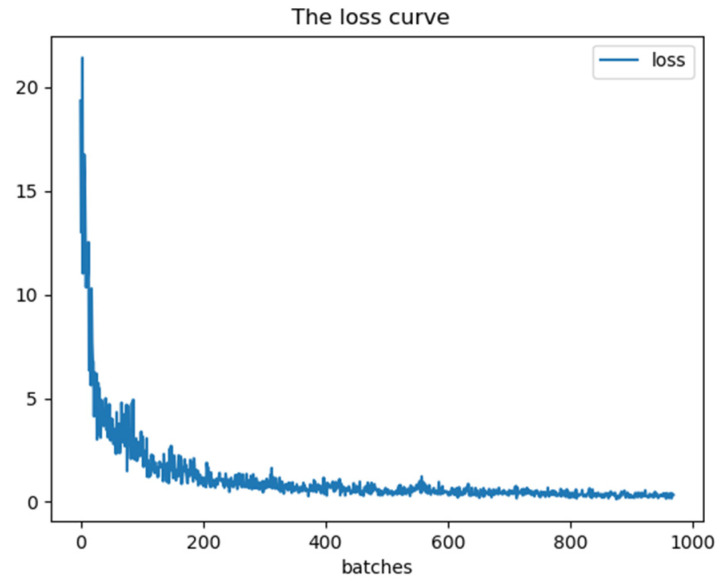
Loss curve in the training process.

The loss curve from the training process is shown in Figure 12.

The loss curve reflects the difference between the recognition result of the model and the actual object category. With the continuous increase of the number of training samples, we can see that the difference is continuously reduced to a stable value, which illustrates that the recognition rate in the training process continues to increase and approaches the theoretical best recognition result.

**Figure 13:**
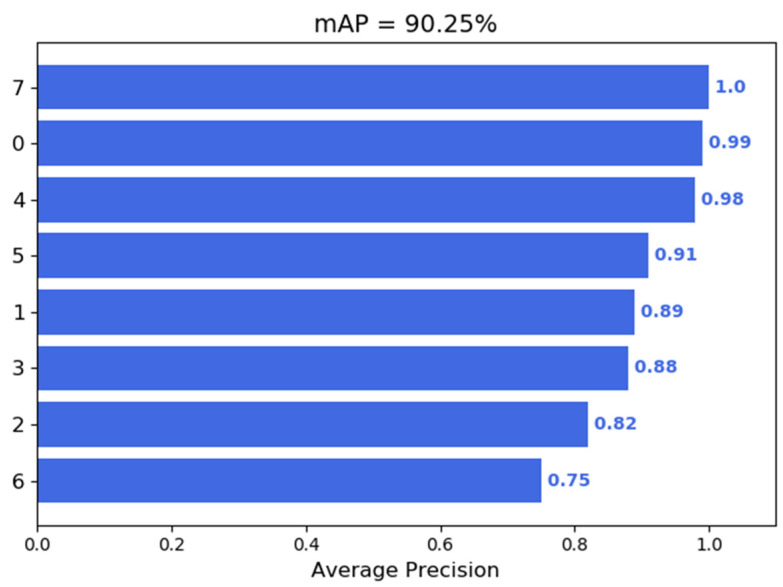
Training set evaluation results.

The performance of the network was verified by checking the relative curve between the registration rate and the recall rate. The statistical results are shown in Figure 13. Besides the category “Other”, the accuracy of the gray category is up to 99%, that of the pink is as the lowest at 75%, the mean average precision (mAP) is about 90.25%. The performance meets metric requirements.

### 4.4. Reasoning Speed and Stability Test

**Figure 14:**
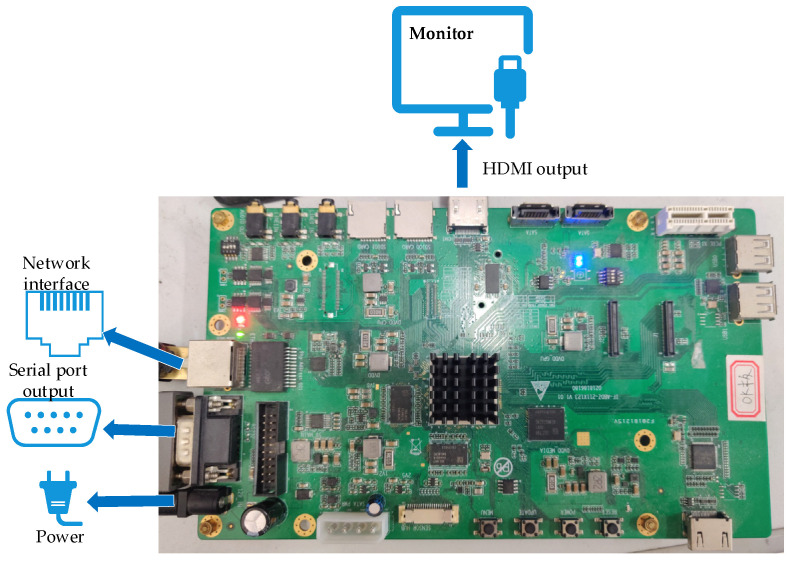
Connection diagram of testing platform.

**Figure 15:**
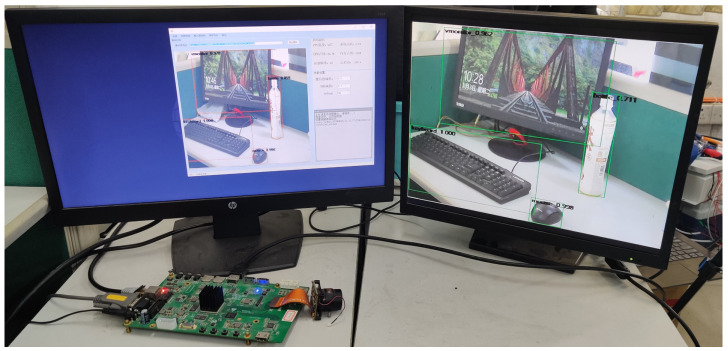
System operating status.

The system’s working status and stability are tested by simulation in the laboratory. The schematic diagram for the equipment connections is shown in Figure 14. The system’s operating status is shown in Figure 15. The output interface of onboard HDMI is connected to the real-time monitoring device on the monitor. The overall recognition console software is operated on the computer display to communicate with the embedded device via internet access.

**Figure 16:**
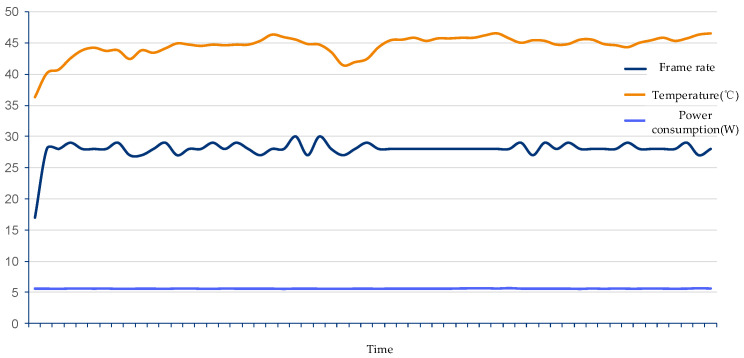
System stability test results.

Finally, in order to test the stability of the solution in this article under long-term operation, this article recorded the system frame rate, temperature and power consumption data within three hours of system operation. The recorded data are shown in Figure 16. The test data show that the frame rate is basically stable at about 28 frames, without significant frame drop during the test time. The chip surface temperature is basically stable after the initial rise. From the above results, the scheme can be used for a long time in actual production and real life, and meet the real-time requirements of this paper.

### 4.5. Results Comparison and Analysis

**Figure 17:**
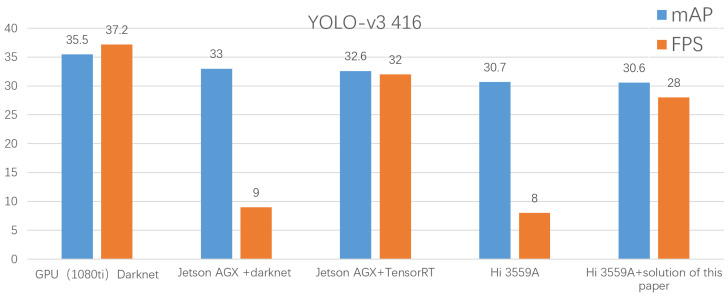
Comparison of network solutions.

In this section, to compare the hardware acceleration scheme of this article and the software acceleration scheme of TensorRT based on the NVIDIA platform, the two platforms simultaneously tested the mAP and FPS of YOLOv3-416 based on the COCO dataset. The comparison results with the existing network in terms of structure accuracy and instantaneity are shown in Figure 17.

The experiment in the above figure reflects the mean average precision (mAP) and frames per second (FPS) of YOLO V3 (IOU = 0.75) on the COCO dataset under different hardware platforms. We chose Nvidia’s embedded edge computing platform Jetson AGX and the server based on 1080Ti were used as references for this solution.

Through the comparison before and after acceleration, we can find that the FPS of the two acceleration schemes was significantly improved after the acceleration, and the FPS after the acceleration has reached the “usable” level. At the same time, the accelerated mAP did not show a significant drop, and it was still within an acceptable range.

**Figure 18:**
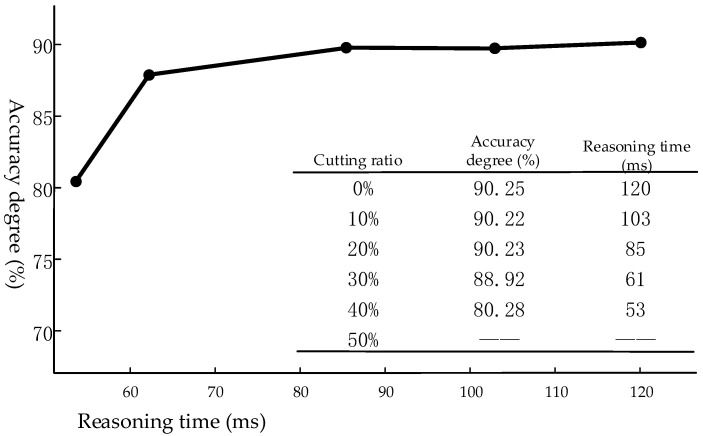
Comparison of experimental results for different cutting ratios.

Even when compared with a server with a 1080Ti card, the mAP and FPS of the solution in this article are not too bad on the COCO dataset. The influence of different cutting results on the accuracy and speed are shown in Figure 18. According to the comparison results, when the pruning amplitude is larger than 20%, the accuracy reduces rapidly with the increase of the pruning ratio. Therefore, a cutting proportion of 20% was used as the final network cutting amplitude.

This paper compared the influence of different deployment schemes and segmentation schemes on the recognition accuracy and frame rate, and the results are shown in Table 4. According to the comparison results, the pipelining technology can ensure the accuracy of the system recognition while greatly reducing the memory consumption. Compared with the double-threaded simultaneous reasoning, the piplining technology greatly reduced the memory consumption and the time for reading disk and output images. This design also limited the network dimension to a multiple of 16, thus greatly improving the reasoning speed of the network.

### 4.6. On-Site Deployment Test

During the on-site deployment test, the system was installed in the kitchen and the data were tested and saved. A total of 1000 images (10 cameras) were randomly selected for the result analysis. The results are shown in Table 5.

**Figure 19:**
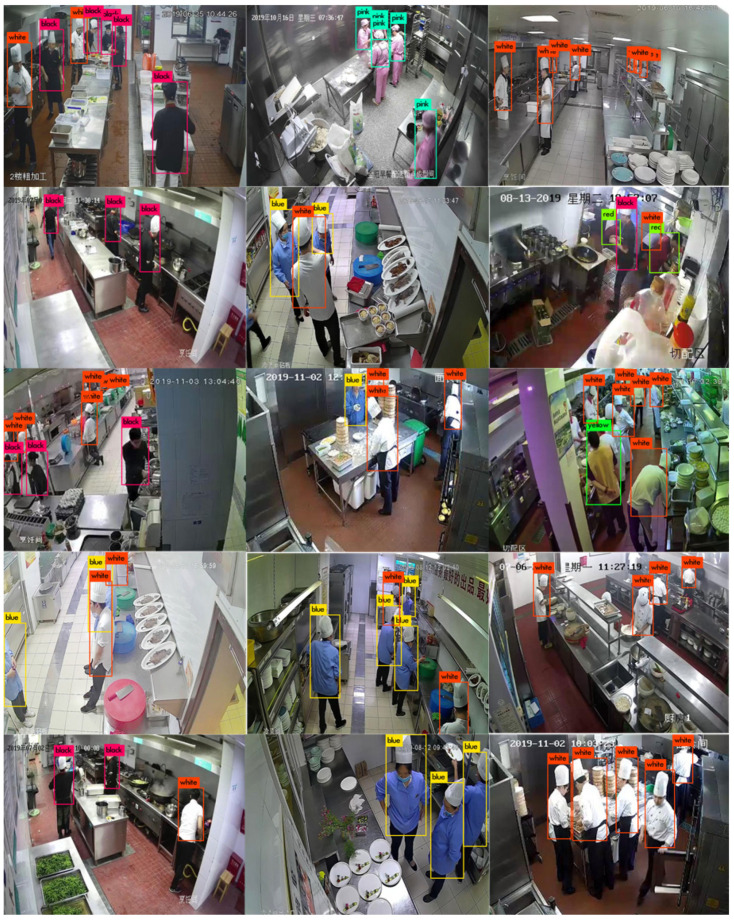
Field test results.

As shown in Table 5, the accuracy rate in most scenes is more than 97%, and the loss rate of some scenes is high, with an average accuracy rate of 98.29%, which basically meets the design requirements. Field test results are shown in Figure 19.

## 5. Conclusions

This paper proposed a scheme for deploying neural networks in embedded devices and applied them to the task of kitchen overalls recognition. This solution significantly reduced the power consumption and equipment cost required for target recognition through the neural network, and further expanded the application range of the neural network. Through the pruning, segmentation, quantification of the network model and the algorithm optimization for the Hi3559A embedded processor hardware, this design realized a good recognition accuracy while increasing the recognition frame rate to about 28 frames, thereby achieving the expected design goal (a recognition frame rate greater than 25). We proved that the embedded platform can complete the recognition task of kitchen overalls through optimization. In the next step, we will try to incorporate some of the latest software acceleration solutions into this solution, and integrate them with the existing hardware acceleration solutions to achieve better acceleration effects.

## Figures and Tables

**Table 1:** Statistics of network calculations.

	Number of Network Layers	Number of Parameters	Calculated Quantity (GFLOPS)	Reasoning Time
Anterior network	78	3,201,760	12.29	32~36
Posterior network	141	58,746,624	20.64	34~40

**Table 2:** Network training environment.

Name	Version No.
CPU Model	Intel core i7-6850k
GPU model	GTX1080Ti ×3
Memory capacity	64 GB
System version	Ubuntu 18.04.5 LTS
CUDA	9.1.85
cud	7.6.5
cafe	1.0.0

**Table 3:** Hyper-parameter settings.

Hyper-Parameter	Value
batch	96
subdivisions	16
decay	0.0005
max_batches	62,000
learning_rate	0.001
policy	Steps
steps	45,000, 50,000, 55,000

**Table 4:** The comparison between different deployment schemes.

Deployment Scheme	Memory Consumption (MB)	Frame Rate (FPS/s)	Precision (mAP)	Overall Power Consumption of the System (W)
Official solutions	81.3	8.9	50.31%	5.0
Double-threaded synchronization	166.1	17.5	50.6%	5.5
Queuing scheme	83.4	27.6	50.8%	5.5
Server deployment (Titan-X)	250	32	51.2%	——

**Table 5:** Record of the field test results.

Camera No.	Identification Error (pcs)	Mislabeling	False Detection	Missed Detection	Accuracy Rate
SXSZBG006	0	0	0	0	100.00%
SXSZBG10	0	0	0	41	95.90%
SXSZBLDJD001	0	0	0	0	100.00%
SXSMHGJD004	0	0	0	19	98.10%
SXXHDJD003	0	0	0	23	97.70%
SXSZGXDJD001	4	0	0	0	99.60%
SXKYMD004	0	0	0	11	98.90%
SXKJJD002	8	0	0	9	98.30%
SXSZYHBG003	9	0	0	21	97.00%
HZCZZX001	0	0	0	26	97.40%
Total	21	0	0	150	98.29%

## Data Availability

Not applicable.
